# Interpersonal Musical Synchronization and Prosocial Behavior in Children: No Effects in a Controlled Field Experiment

**DOI:** 10.3389/fpsyg.2021.784255

**Published:** 2021-12-10

**Authors:** Janina Baier, Clemens Wöllner, Anna Wolf

**Affiliations:** Institute for Systematic Musicology, University of Hamburg, Hamburg, Germany

**Keywords:** replication, social bonding, altruism, kindergarten children, singing, music, theory of mind

## Abstract

Prosocial effects of music have recently attracted increased attention in research and media. An often-cited experiment, carried out by Kirschner and Tomasello in 2010 under laboratory conditions, found that children at the age of four years were more willing to help each other after they had engaged in synchronous musical activities. The aim of the current study was to replicate this research under controlled field conditions in the children's social environment, and to disentangle the musical synchronization effect by introducing a verbal interaction (singing together) and a motor interaction (tapping together) task, contrasted by an asynchronous control condition. In a between-participants design, no effects of musical synchronization nor the children's gender were found. Furthermore, age was not related to prosocial behavior. Explanations are systematically discussed, yet it remains possible that the original effect found in 2010 might be overestimated and less consistently reproducible as previously assumed.

## Introduction

Music as a cultural technique may have strong effects on everyone who engages with it by singing, playing an instrument, listening or dancing. Plainly, without such effects, it would not have co-evolved in all cultures alongside language. A highly influential and widely cited experiment showed that kindergarten children were more prone to help a fellow child in a misfortunate situation if both had previously engaged in a synchronized musical exercise (Kirschner and Tomasello, 2010). In the current replication study, we re-examined this effect by placing the same experiment in the children's daily-life social environment, and by differentiating between the synchronization conditions.

This line of research can be seen within the wider topic of musical transfer effects. Making music or listening to music should result in non-musical benefits in domains such as language ability (Swaminathan and Schellenberg, 2020), or spatial reasoning as a subcomponent of intelligence. The latter, notoriously entitled “Mozart effect,” has stirred a controversy in the research community and beyond, as several studies could not replicate the initial outcome (cf. Pietschnig et al., 2010). Research on music's social effects, on the other hand, has hardly been questioned. Studies have emphasized, for instance, the importance of singing for feelings of belonging and social bonding (Bailey and Davidson, 2001; Kreutz, 2014; Pearce et al., 2015). Given the manifold coordination tasks that need to be accomplished for joint music making in professional or lay musical formations (cf. Wöllner and Keller, 2017), one could assume that this translates into coordinated or more “social” behavior even outside music making (Savage et al., 2020).

Altruistic behavior in relation to music may have various reasons. Both calming and stimulating music were found to improve the mood and subsequently to increase helping behavior (Fried and Berkowitz, 1979; North et al., 2004). Furthermore, music training has been shown to be related to higher empathy (Rabinowitch et al., 2013) and prosocial skills in children (Schellenberg et al., 2015). One of the musical mechanisms for increased prosocial behavior could lie in the need to synchronize with others. Research has shown that synchronously timed body movements lead to higher ratings of liking and affiliation (Hove and Risen, 2009) and subsequently to higher compliance (Wiltermuth and Heath, 2009), compassion and altruism (Valdesolo and DeSteno, 2011).

In the study replicated here, Kirschner and Tomasello (2010) asked pairs of 4-year old children to take part in a task that confronts one of the children with a problem situation. The other child then has to decide to what extent they are willing to help. Those children that took part in a musical synchronization game before the problem situation demonstrated significantly higher prosocial behavior compared to children in the non-musical control group. In other words, singing and dancing together resulted in more helping behavior. Kirschner and Tomasello believe that music may both have enhanced the children's mood as well as their social bonding *via* interpersonal synchrony. It should be noted that children had known each other from their kindergartens, but took part in a laboratory setting outside their daily-life environment. The children's gender influenced results, with pairs of girls being more helpful to each other compared to pairs of boys. Other research has shown that children at this age have developed a sense for social norms (Schmidt et al., 2016) and empathic behavior (Grosse Wiesmann et al., 2018; Kammermeier and Paulus, 2018); children younger than that are still developing a notion of theory of mind, with 3 years old being a critical age (Wellman, 1992).

Since the musical intervention in Kirschner and Tomasello (2010) comprised both a musical-verbal and musical-motor component, it remains an open question as to whether the prosocial effects are primarily related to joint singing (e.g., Bailey and Davidson, 2001; Kreutz, 2014; Welch et al., 2014; Pearce et al., 2015; Good and Russo, 2016; Stewart and Lonsdale, 2016), or rather interpersonal motor synchrony (e.g., Wiltermuth and Heath, 2009; Valdesolo and DeSteno, 2011; Cirelli et al., 2014). If both musical components are at play, which one leads to stronger prosocial effects?

### Aims

The current study investigated the respective impact of verbal and motor musical synchronization in kindergarten children. The intervention and spontaneous helping test were adapted from Kirschner and Tomasello (2010). It was hypothesized that

1) A child in one of two musical conditions, compared to a non-musical control condition, would be more willing to help the other child in a given task. There would be differences between musical-verbal (singing) and musical-motor (tapping) conditions.
2) Based on previous research, the children's gender should influence the results.
3) Age was expected to have an impact on findings, with older children engaging in more prosocial behavior.

The main aim was to replicate and specify the effects on prosocial behavior following a musical intervention.

## Methods

Eighty-four kindergarten children (aged 3–6 years, *M* = 4.24 years; 52 male, 32 female) took part in the study. Children were tested in four kindergartens in pairs of two such that their age and gender were matched. The intervention material was identical to that of the original study, where the children sang and walked in synchrony to a frog song in the musical condition. Before taking part, pairs of children were randomly assigned to one of three experimental conditions:

a) musical-verbal (i.e., singing a frog song),
b) musical-motor (i.e., striking a wooden croaking frog), or
c) non-musical control (i.e., speaking the lyrics of the frog song without an even meter).

There was no significant difference in age between the three conditions [*F*_(2, 39)_ = 1.56, *p* = 0.22, η^2^ = 0.074]. Because of the random condition assignment, in a) there were eight girls and 20 boys, whereas for both (b) and (c) there were 12 girls and 16 boys each; these frequencies were not significantly different [χ^2^_(2)_ = 1.62, *p* = 0.446]. The study was approved by the Ethics Committee of the Faculty of Humanities, University of Hamburg, and the children's parents provided informed consent prior to the experimental field study.

**Figure 1:**
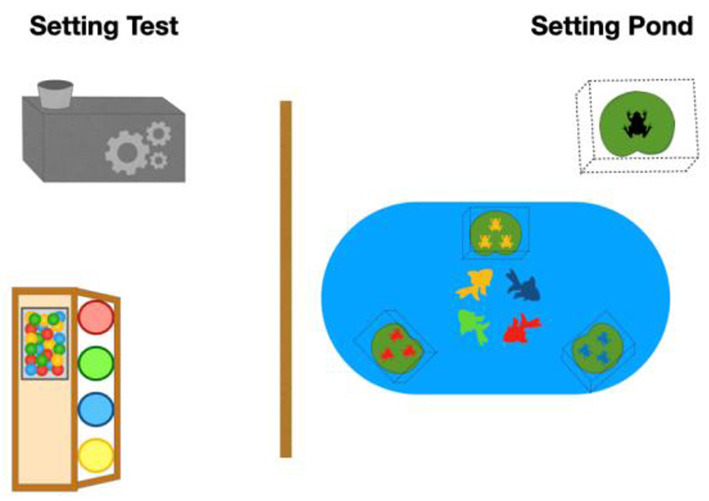
The setting of the field experiment.

Children were asked by the experimenter if they would enjoy taking part. One of the three conditions (a), (b), or (c) was then introduced to the children by the same experimenter, so the children could simply reproduce her actions at the fish pond setting (Figure 1). The task was repeated three times before the actual helping test started, in which the experimenter introduced the children to the feeding station for the fishes, where the children had to transport the “fish food” in colored tubes to a “grinder.” The helping test aimed at assessing if and for how long one of the children assisted the other one in a deliberate problem situation (i.e., the tube broke when moving “fish food” to the “grinder”). Before the experiment, it was randomly assigned which child in the pair should bring the fish food to the grinder. The behavior of the other child was categorized into

- A: actively helping until the problem was solved;
- B1: waiting;
- B2: briefly helping, then leaving;
- B3: waiting, then leaving;
- C: immediately leaving, neither helping nor waiting.

In order to avoid experimenter effects, the different types of behaviors were recorded by an assistant to the experimenter, who did not interact with the children before in the musical or non-musical intervention phase. The “fish food station” was separated from the pond by a folding screen, so that the experimenter and the children could not see each other during the helping test. The assistant had received guidance instructions about how to observe the children's behavior, and was seated in distance from the children without making them aware that their responses were noted down. Frequencies of behaviors were subsequently statistically compared across groups using chi-square tests. Since only the behavior of the (non-)helping child in the pair was analyzed, statistics were based on *n* = 42 (out of the total of 84 children).

## Results

**Figure 2:**
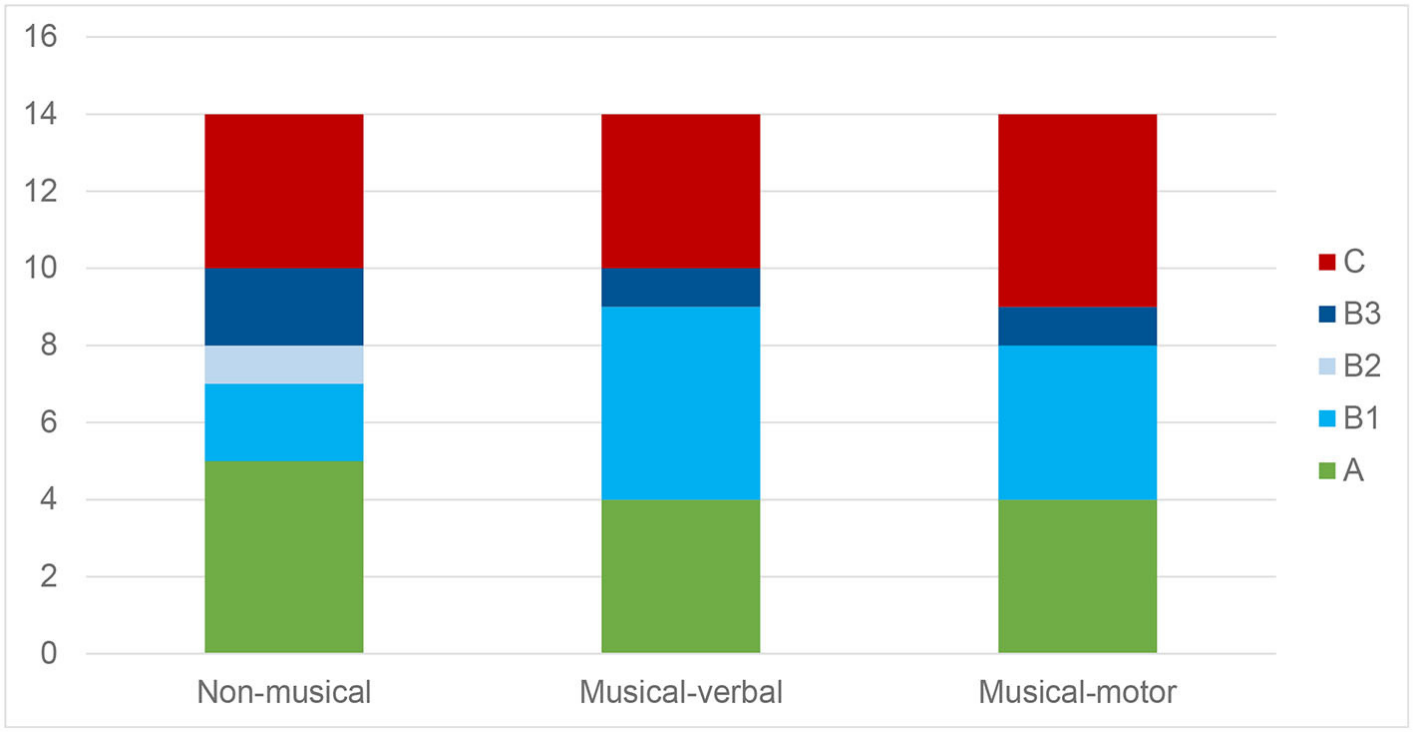
Frequency of prosocial behavior in the three stimulation conditions.

The three experimental conditions did not lead to significant differences in children's behavior [χ^2^_(8)_ = 4.08, *p* = 0.850; Figure 2]. Strictly following the analyses in Kirschner and Tomasello (2010), numbers of children who actively helped (category A responses) did not significantly differ from the control condition either [both χ^2^_(1)_ = 0.11, *p* = 0.739]. In other words, the children's willingness to engage in prosocial behavior was not influenced by a prior intervention that included musical-verbal or musical-motor synchronization with each other, in comparison to a verbal control condition with no synchronization.

**Figure 3:**
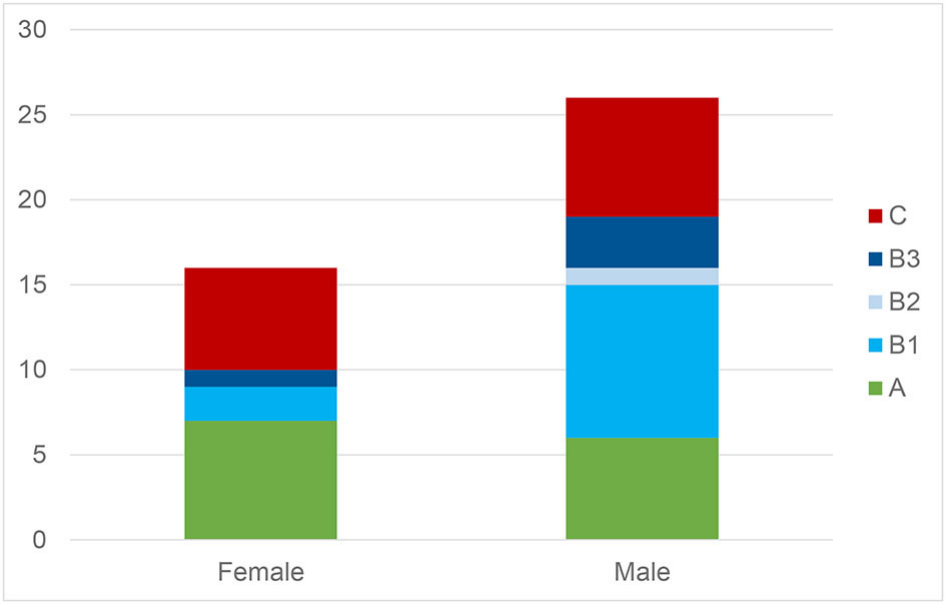
Frequency of prosocial behavior in relation the children's gender.

Gender norms did also not significantly influence types of behavior [χ^2^_(4)_ = 4.48, *p* = 0.345; Figure 3]. Girls or boys did not actively help more compared to the respective other gender [χ^2^_(1)_ = 0.08, *p* = 0.782].

Finally, effects of age were analyzed with a non-parametric Spearman correlation on the five helping options in ascending order, which resulted in no significant relationship [*r*_S_(42)_ = −0.08, *p* = 0.604]. Based on assumptions that 3-year olds may less or not be able to empathize with others, the statistical tests above were re-analyzed without the eight 3-year old children, again resulting in no effect for condition [χ^2^_(8)_ = 4.31, *p* = 0.829] or gender [χ^2^_(4)_ = 3.00, *p* = 0.558].

## Discussion

Children who had engaged in musical activities did not help other children more compared to a control group. There are several explanations and potential limitations for the failure to replicate the prosocial effects as found by Kirschner and Tomasello (2010), as we discuss in the following. It should be noted that our aim had been to disentangle the musical synchronization effect and not to falsify the original hypotheses and findings. We believe that presenting the null effects in the replication provides a fuller picture and complements this line of research in a necessary way.

First, the main difference between the original research and our replication study was the differentiation between musical-motor (tapping) and musical-verbal (singing) conditions. Both are joint activities that comprise interpersonal synchronization, which was found to increase prosocial behavior and altruism in previous research (e.g., Wiltermuth and Heath, 2009; Valdesolo and DeSteno, 2011). Nevertheless, while social effects of singing may primarily be mood-related (Bailey and Davidson, 2001; Kreutz, 2014; Pearce et al., 2015), synchronous tapping together may increase liking and social connectedness (Hove and Risen, 2009). Both conditions were expected to result in more prosocial behavior compared to the non-musical, asynchronous control condition. It may well be that for young children, only the combination of singing and dancing causes the significant effects as found in Kirschner and Tomasello (2010). The same holds true for the gender effects that were not present in the replication. We have used the same song as in the original study, where it led to clear effects between conditions. Further replications might use different music or motor tasks that facilitate children's engagement and synchronization.

Second, our replication further differed in that data were collected in a familiar room of the children's kindergarten (Kirschner and Tomasello: in a laboratory). Furthermore, the experimenter was still in the room during the helping test, but separated by a room-divider. Since children were highly absorbed in the task, they should have helped each other regardless of others, particularly in their daily social environment. Perhaps the unfamiliar room in the original study caused a stronger feeling of connectedness that was still enforced by the musical task, which in daily-life situations is less pronounced. Controlled field experiments are needed to provide valid results particularly with children.

Third, children's age may have influenced results. There is some controversy about the development of empathy and theory of mind in children (Grosse Wiesmann et al., 2018; Kammermeier and Paulus, 2018), with some researchers stating that 3 years of age are critical for development (Wellman, 1992). In the original study, all children were 4 years old. In our replication, there was no correlation with age, and statistical re-analysis of main results without the 3-year old children did not change results. Other research has shown effects of musical training on empathy development in children (Rabinowitch et al., 2013), and relations between music and empathic responses have long been assumed (cf. Wöllner, 2017). It may therefore be plausible that long-term musical training rather than only a short exposure should enhance prosocial effects.

Finally, effects might primarily be related to improved mood (cf. North et al., 2004). Great care was laid on having exactly the same stimulating and engaging interaction of the experimenter with all children, regardless of their group and condition. While we firmly believe that the same was done in the original study, mood could be key to children's helping behavior and should be further investigated in controlled field studies. In other words, it cannot be ruled out that the original effects as found in a laboratory situation, following a short-term musical intervention, were potentially to some extent overstated.

## Conclusion

This study did not replicate the striking effects of musical synchronization on prosocial behavior as found by Kirschner and Tomasello (2010). While the present experiment split the musical intervention into a verbal and a motor component, these synchronous musical conditions should still have led to more prosocial behavior compared to the non-musical control condition. In contrast to the original study, children were tested in their daily-life social environment, so it remains plausible that the effects found in 2010 might not be as consistently reproducible in non-laboratory conditions as previously assumed.

## Data Availability Statement

The raw data and furter material are included in the article/Supplementary Material, further inquiries can be directed to the corresponding author/s.

## Ethics Statement

The study involving human participants was reviewed and approved by the Ethics Committee of the Faculty of Humanities (EKGW), University of Hamburg. Written informed consent to participate in this study was provided by the participants' legal guardian/next of kin.

## Author Contributions

JB collected the data. JB and CW analyzed the data and searched literature. AW prepared the figures and a conference presentation. CW wrote the first version of the current manuscript. All authors discussed and agreed to the final version.

## Conflict of Interest

The authors declare that the research was conducted in the absence of any commercial or financial relationships that could be construed as a potential conflict of interest.

## Publisher's Note

All claims expressed in this article are solely those of the authors and do not necessarily represent those of their affiliated organizations, or those of the publisher, the editors and the reviewers. Any product that may be evaluated in this article, or claim that may be made by its manufacturer, is not guaranteed or endorsed by the publisher.

## References

[B1] Bailey B. A. Davidson J. W. (2001). “Emotional, social, and cognitive enrichment through participation in group singing: Interviews with members of a choir for homeless men,” in Proceedings of The Phenomenon of Singing International Symposium III, 24–32. Available online at: https://journals.library.mun.ca/ojs/index.php/singing/article/view/625 (accessed November 22, 2021).

[B2] Cirelli L. K. Einarson K. M. Trainor L. J. (2014). Interpersonal synchrony increases prosocial behavior in infants. Dev. Sci. 17:1003. 10.1111/desc.12193 25513669

[B3] Fried R. Berkowitz L. (1979). Music hath charms and can influence helpfulness. J. Appl. Soc. Psychol. 9, 199–208. 10.1111/j.1559-1816.1979.tb02706.x

[B4] Good A. Russo F. A. (2016). Singing promotes cooperation in a diverse group of children. Soc. Psychol. 47, 340–344. 10.1027/1864-9335/a000282

[B5] Grosse Wiesmann C. Friederici A. D. Disla D. Steinbeis N. Singer T. (2018). Longitudinal evidence for 4-year-olds' but not 2- and 3-year-olds' false beliefs related action anticipation. Cogn. Dev. 46, 58–68. 10.1016/j.cogdev.2017.08.007 30147231 PMC6103291

[B6] Hove M. J. Risen J. L. (2009). It's all in the timing: interpersonal synchrony increases affiliation. Soc. Cogn. 27, 949–961. 10.1521/soco.2009.27.6.949

[B7] Kammermeier M. Paulus M. (2018). Do action-based tasks evidence false-belief understanding in young children? Cogn. Dev. 46, 31–39. 10.1016/j.cogdev.2017.11.004

[B8] Kirschner S. Tomasello M. (2010). Joint music making promotes prosocial behavior in 4-year-old children. Evol. Hum. Behav. 31, 354–364. 10.1016/j.evolhumbehav.2010.04.004

[B9] Kreutz G. (2014). Does singing facilitate social bonding? Music Med. 6, 51–60. 10.47513/mmd.v6i2.180

[B10] North A. C. Tarrant M. Hargreaves D. J. (2004). The effects of music on helping behavior: a field study. Environ. Behav. 36, 266–275. 10.1177/0013916503256263

[B11] Pearce E. Launay J. Dunbar R. (2015). The ice-breaker effect: singing mediates fast social bonding. Royal Soc. Open Sci. 2:150221. 10.1098/rsos.150221 26587241 PMC4632513

[B12] Pietschnig J. Voracek M. Formann A. K. (2010). Mozart effect-Shmozart effect: a meta-analysis. Intelligence 38, 314–323. 10.1016/j.intell.2010.03.001

[B13] Rabinowitch T. Cross I. Burnard P. (2013). Long-term musical group interaction has a positive influence on empathy in children. Psychol. Music 41, 484–498. 10.1177/0305735612440609

[B14] Savage P. Loui P. Tarr B. Schachner A. Glowacki L. Mithen S.. (2020). Music as a coevolved system for social bonding. Behav. Brain Sci. 2020, 1–36. 10.31234/osf.io/qp3st 32814608

[B15] Schellenberg E. Corrigall K. Dys S. Malti T. (2015). Group music training and children's prosocial skills. PLoS ONE. 10:e0141449. 10.1371/journal.pone.0141449 26506414 PMC4624672

[B16] Schmidt M. Butler L. Heinz J. Tomasello M. (2016). Young children see a single action and infer a social norm: promiscuous normativity in 3-year-olds. Psychol. Sci. 27, 1360–1370. 10.1177/0956797616661182 27634004

[B17] Stewart N. Lonsdale A. (2016). It's better together: the psychological benefits of singing in a choir. Psychol. Music 44, 1240–1254. 10.1177/0305735615624976

[B18] Swaminathan S. Schellenberg E. G. (2020). Musical ability, music training, and language ability in childhood. J. Exp. Psychol. Learn. Mem. Cogn. 46, 2340–2348. 10.1037/xlm0000798 31750723

[B19] Valdesolo P. DeSteno D. (2011). Synchrony and the social tuning of compassion. Emotion 11, 262–266. 10.1037/a0021302 21500895

[B20] Welch G. Himonides E. Saunders J. Papageorgi I. Sarazin M. (2014). Singing and social inclusion. Front. Psychol. 5:803. 10.3389/fpsyg.2014.00803 25120514 PMC4114289

[B21] Wellman H. M. (1992). The Child's Theory of Mind. Cambridge, MA: MIT Press.

[B22] Wiltermuth S. Heath C. (2009). Synchrony and cooperation. Psychol. Sci. 20, 1–5. 10.1111/j.1467-9280.2008.02253.x 19152536

[B23] Wöllner C. (2017). Audience responses in the light of perception-action theories of empathy, in Music and Empathy, eds King E. Waddington C. (London: Routledge), 139–156. 10.4324/9781315596587-8

[B24] Wöllner C. Keller P. (2017). Music with others: ensembles, conductors, and interpersonal coordination, in The Routledge Companion to Music Cognition, eds Ashley R. Timmers R. (London: Routledge), 313–324. 10.4324/9781315194738-26

