# Supplementary material for: Interpersonal Musical Synchronization and Prosocial Behavior in Children: No Effects in a Controlled Field Experiment
Source: Front Psychol. 2021 Dec 10;12:784255. doi: 10.3389/fpsyg.2021.784255 (PMC8707737; doi:10.3389/fpsyg.2021.784255)

Supplementary material: ***Interpersonal musical synchronization and prosocial behavior in children: No effects in a controlled field experiment***

Janina Baier, Clemens Wöllner, and Anna Wolf
University of Hamburg, Hamburg, Germany

Raw data

**Condition**[1]	**Gender**[2]	**Age (years)**	**Helping behavior**[3]
1	m	5	1
1	m	6	5
1	f	4	5
1	f	4	1
1	m	5	4
1	m	3	4
1	m	4	2
1	f	5	5
1	m	4	1
1	f	4	1
1	m	5	1
1	f	3	5
1	f	5	2
1	m	4	3
2	f	4	2
2	f	5	1
2	m	3	5
2	m	3	5
2	m	5	2
2	m	5	2
2	f	4	1
2	m	4	5
2	m	3	4
2	m	3	2
2	m	4	1
2	f	4	5
2	m	4	1
2	m	4	2
3	f	5	5
3	f	3	5
3	f	6	4
3	f	4	1
3	m	4	1
3	f	4	1
3	m	5	5
3	m	3	2
3	m	5	2
3	f	5	1
3	m	5	5
3	m	4	5
3	m	4	2
3	m	5	2

[1] Numbers in the table indicate: 1 = non-musical control, 2 = musical-verbal, 3 = musical-motor
[2] f = female, m = male
[3] Numbers for response categories indicate: 1 = A (actively helping), ..., 5 = C (immediately leaving, neither helping nor waiting). See main article for further detail.

The musical material (“frog song”) can be found in the supplementary material of the original study (Kirschner & Tomasello, 2010).

Photos of the equipment (copyright: J. Baier): The pond, the fish food station with the manipulated tubes, and the grinder (see Fig. 1).

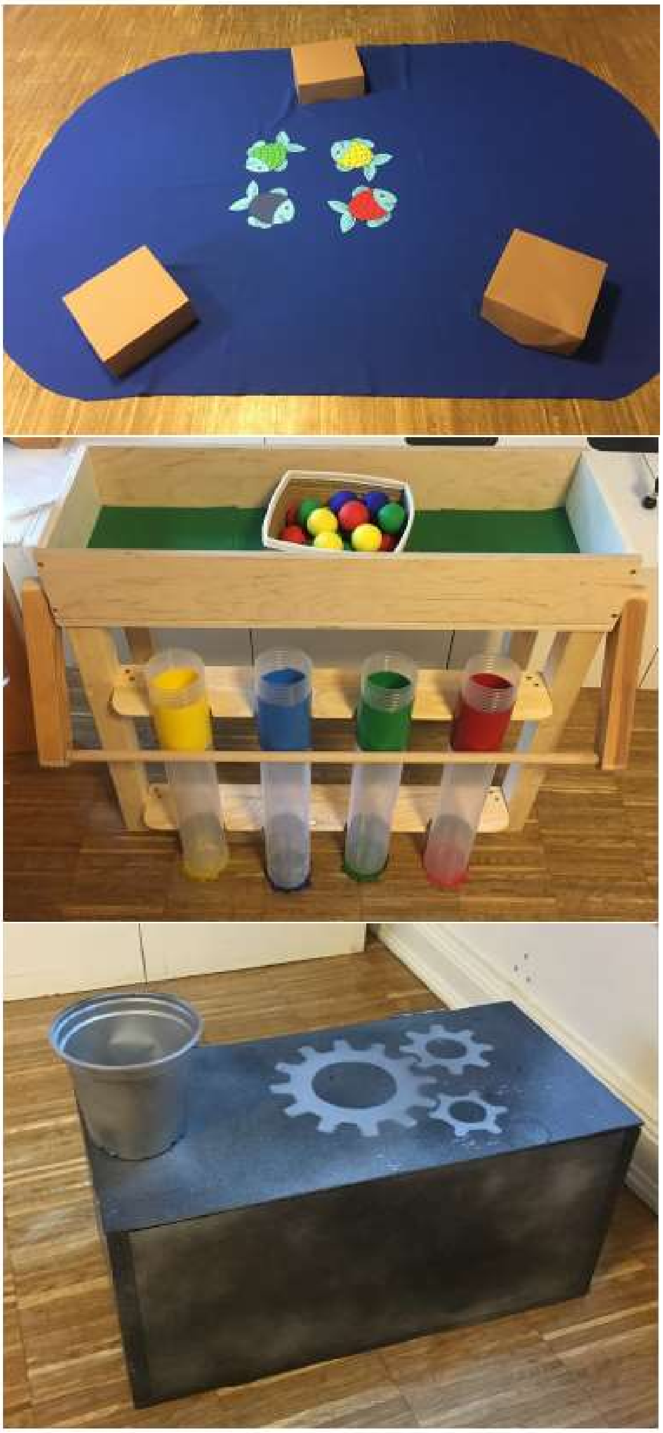

Supplement: Supplementary file 1 [file Data_Sheet_1.pdf]
